# Genotype-Phenotype Correlations in *RP1*-Associated Retinal Dystrophies: A Multi-Center Cohort Study in JAPAN

**DOI:** 10.3390/jcm10112265

**Published:** 2021-05-24

**Authors:** Kei Mizobuchi, Takaaki Hayashi, Noriko Oishi, Daiki Kubota, Shuhei Kameya, Koichiro Higasa, Takuma Futami, Hiroyuki Kondo, Katsuhiro Hosono, Kentaro Kurata, Yoshihiro Hotta, Kazutoshi Yoshitake, Takeshi Iwata, Tomokazu Matsuura, Tadashi Nakano

**Affiliations:** 1 Department of Ophthalmology, The Jikei University School of Medicine, 3-19-18, Nishi-shimbashi, Minato-ku, Tokyo 105-8471, Japan; taka@jikei.ac.jp (T.H.); tnakano@jikei.ac.jp (T.N.); 2 Department of Ophthalmology, Katsushika Medical Center, The Jikei University School of Medicine, 6-41-2 Aoto, Katsushika-ku, Tokyo 125-8506, Japan; 3 Department of Ophthalmology, Nippon Medical School Chiba Hokusoh Hospital, 1715 Kamagari, Inzai, Chiba 270-1694, Japan; oishinoriko@nms.ac.jp (N.O.); 4d.freewill27@gmail.com (D.K.); shuheik@nms.ac.jp (S.K.); 4 Department of Genome Analysis, Institute of Biomedical Science, Kansai Medical University, 2-5-1 Shinmachi, Hirakata, Osaka 573-1010, Japan; higasa@genome.med.kyoto-u.ac.jp; 5 Department of Ophthalmology, University of Occupational and Environmental Health, 1-1, Iseigaoka, Yahatanishi-ku Kitakyushu-shi, Fu-kuoka 807-8555, Japan; futataku_uoeh@yahoo.co.jp (T.F.); kondohi@med.uoeh-u.ac.jp (H.K.); 6 Department of Ophthalmology, Hamamatsu University School of Medicine, 1-20-1, Handayama, Higashi-ku, Shizuoka, Hamamatsu 431-3192, Japan; hosono@hama-med.ac.jp (K.H.); 41222975@hama-med.ac.jp (K.K.); hotta@hama-med.ac.jp (Y.H.); 7 National Institute of Sensory Organs, National Hospital Organization Tokyo Medical Center, 2-5-1 Higashigaoka, Meguro-ku, Tokyo 152-8902, Japan; yoshitake.kazutoshi@kankakuki.go.jp (K.Y.); takeshi.iwata@kankakuki.go.jp (T.I.); 8 Department of Laboratory Medicine, The Jikei University School of Medicine, 3-19-18, Nishi-shimbashi, Minato-ku, Tokyo 105-8471, Japan; matsuurat@jikei.ac.jp

**Keywords:** *RP1* gene, next generation sequencing, retinitis pigmentosa, cone-rod dystrophy, inherited retinal disease

## Abstract

Background: Little is known about genotype–phenotype correlations of *RP1*-associated retinal dystrophies in the Japanese population. We aimed to investigate the genetic spectrum of *RP1* variants and provide a detailed description of the clinical findings in Japanese patients. Methods: In total, 607 patients with inherited retinal diseases were examined using whole-exome/whole-genome sequencing (WES/WGS). PCR-based screening for an *Alu* element insertion (c.4052_4053ins328/p.Tyr1352AlafsTer9) was performed in 18 patients with autosomal-recessive (AR)-retinitis pigmentosa (RP) or AR-cone dystrophy (COD)/cone-rod dystrophy (CORD), including seven patients with heterozygous *RP1* variants identified by WES/WGS analysis, and 11 early onset AR-RP patients, in whom no pathogenic variant was identified. We clinically examined 25 patients (23 families) with pathogenic *RP1* variants, including five patients (five families) with autosomal-dominant (AD)-RP, 13 patients (11 families) with AR-RP, and seven patients (seven families) with AR-COD/CORD. Results: We identified 18 pathogenic *RP1* variants, including seven novel variants. Interestingly, the *Alu* element insertion was the most frequent variant (32.0%, 16/50 alleles). The clinical findings revealed that the age at onset and disease progression occurred significantly earlier and faster in AR-RP patients compared to AD-RP or AR-COD/CORD patients. Conclusions: Our results suggest a genotype–phenotype correlation between variant types/locations and phenotypes (AD-RP, AR-RP, and AR-COD/CORD), and the *Alu* element insertion was the most major variant in Japanese patients with *RP1*-associated retinal dystrophies.

## 1. Introduction

Retinitis pigmentosa (RP) is a heterogeneous group of inherited retinal disease characterized by night blindness, progressive visual field loss, and eventually loss of visual acuity. RP can be classified into non-syndromic and syndromic RP [1]. The inheritance pattern of non-syndromic RP shows autosomal dominant (AD), autosomal recessive (AR), X-linked, sporadic/isolated, mitochondrial, and digenic inheritance [2,3,4]. To date, over 80 genes has been reported as cause of non-syndromic RP. The genes associated with RP play various important roles including phototransduction cascade, vitamin A metabolism, structural or cytoskeletal, signaling, cell–cell interaction/synaptic interaction, RNA intron-splicing factors, trafficking of intracellular proteins, maintenance of cilia/ciliated cells, pH regulation, phagocytosis, and yet unknown function [5]. The retinitis pigmentosa 1 (*RP1*) gene consists of 4 exons and encodes a 2156-amino-acid photoreceptor-specific microtubule-associated protein containing two doublecortin (DCX) domains (amino acid residues 36 to 118 and 154 to 233) [6,7], via which the RP1 protein interacts with microtubules [8]. In addition, the RP1 protein contains a region homologous with the Drosophila melanogaster bifocal (BIF) protein (amino acid residues 486 to 635), which is required for normal photoreceptor morphogenesis [6]. The RP1 protein is localized on the connecting cilium and axoneme of both rod and cone photoreceptors [9,10], and is involved in the transport of proteins between the inner and outer segments of photoreceptors, cilial structure maintenance, and stabilization of disc membranes in the outer segment [9,11]. The *RP1* gene is one of eight causative genes, including *BEST1*, *NR2E3*, *NRL*, *RDH12*, *RHO*, *RPE65*, and *SAG*, all of which have been associated with both AD-RP and AR-RP [12,13]. The prevalence of AD-RP and AR-RP associated with *RP1* is approximately 5.5% of all AD-RP and 4.5% of all AR-RP in European populations [14,15]. In the AD-RP phenotype, all truncated *RP1* variants are located within a defined hotspot region in exon 4 between amino residues 500 and 1053 [16,17]. In contrast, most biallelic variants, which are located around the N- or C-terminals of the *RP1* gene, are associated with the AR-RP phenotype [18]. Patients with AR-RP generally exhibit much more severe phenotypes than patients with AD-RP [19]. A recent study revealed that an *Alu* element insertion, leading to the c.4052_4053ins328 (p.Tyr1352AlafsTer9) variant, has been frequently found in Japanese patients with hereditary retinal degenerations, demonstrating that 6 of the 331 patients (1.8%, 12/662 alleles) had the *Alu* element insertion homozygously [20]. However, clinical phenotypes of the hereditary retinal degenerations have not been determined in that study [20]. More recent studies have reported the clinical and genetic features associated with the *Alu* element insertion in Japanese patients with AR-RP [20,21,22]. In addition, the *RP1* gene has been reported as a cause of the AR-cone dystrophy (COD)/cone-rod dystrophy (CORD) phenotype [20,23,24,25]. Verbakel et al. reported detailed clinical findings of 11 patients with AR-COD/CORD [25]. However, little is known about the longitudinal findings of patients with AR-COD/CORD, and the genotype–phenotype correlations in *RP1*-associated retinal dystrophies in the Japanese population. Therefore, we aimed to clarify the genotype–phenotype correlations and describe the detailed clinical findings of *RP1*-associated retinal dystrophies.

## 2. Materials and Methods

### 2.1. Ethics Statement

The study protocol was approved by the Institutional Review Boards of The Jikei University School of Medicine (approval number, 24–231 6997), Nippon Medical School Chiba Hokusoh Hospital (approval number, 27–02), Hamamatsu University School of Medicine (approval number, 14–040), University of Occupational and Environmental Health (approval number, H29–03), and the National Hospital Organization Tokyo Medical Center (approval number, R18–029). The protocol adhered to the tenets of the Declaration of Helsinki, and informed consent was obtained from the participants.

### 2.2. Molecular Genetic Study

#### 2.2.1. Next-Generation Sequencing

We studied a total of 607 patients with inherited retinal diseases (IRDs) from 440 families, including 475 patients from 344 families at The Jikei University Hospital, 100 patients from 67 families at Nippon Medical School Chiba Hokusoh Hospital, 23 patients from 22 families at University of Occupational and Environmental Health Hospital, and 9 patients from 7 families at Hamamatsu University Hospital, who underwent whole-exome sequencing (WES) or whole-genome sequencing (WGS) analysis. Details of the WES and WGS methodologies have been described previously [26,27,28,29,30,31]. We evaluated the pathogenicity of the obtained *RP1* variants according to the frequency using the Human Gene Mutation Database Professional (HGMD, http://www.hgmd.cf.ac.uk/, accessed on January, 2020), Genome Aggregation Database (gnomAD, https://gnomad.broadinstitute.org/, accessed on 29 October 2020), inheritance pattern, phenotype, and American College of Medical Genetics standards (ACMG) criteria.

#### 2.2.2. Screening for *Alu* Element Insertion

The *Alu* element insertion could not be efficiently detected by the exon capture process of WES. Therefore, after WES/WGS analysis, we performed polymerase chain reaction (PCR)-based screening for the *Alu* element insertion in 18 patients from 18 families with AR-RP or AR-COD/CORD who carried heterozygous *RP1* variants identified by WES/WGS analysis, and 11 patients with early onset AR-RP from 9 families, in whom no pathogenic variant was identified by WES analysis. The following primer set was used for the detection of *Alu* element insertion: exon 4 forward primer 5′-TGTGCTCAAAAGGA-GAACCATAC-3′ and reverse primer 5′-TCCTGAAACTTCCTTAGTGAAC-3′. The *Alu* element insertion was confirmed by size differences (expected sizes of the *Alu* element insertion and a wild type are 675 bp and 347 bp, respectively) in PCR products on electrophoresis.

### 2.3. Clinical Examinations

We performed comprehensive ophthalmic examinations, including decimal best-corrected visual acuity (BCVA) measurement, fundus photography, fluorescein angiography (FA), fundus autofluorescence imaging (FAF) using a Spectralis HRA (Heidelberg Engineering, Heidelberg, Germany) and/or Optos 200Tx/California Ultra-widefield Retinal Imaging System (Optos, Dunfermline, UK), optical coherence tomography (OCT; Carl Zeiss Meditec AG, Dublin, CA, USA), and Goldmann perimetry (GP; Haag Streit, Bern, Switzerland). The visual field areas of I-4e and V-4e isopters were measured using FIJI/ImageJ software (available at https://fiji.sc, accessed on 1 December 2019). Full-field electroretinography (ERG) was recorded in accordance with the protocols of the International Society for Clinical Electrophysiology of Vision [32] by using a built-in light-emitting diode electrode (LE-4000; Tomey, Nagoya, Japan) or a Ganzfeld dome with an EOG-ERG Ganzfeld stimulator (Electrophysiology system; LACE Elettronica). The detailed procedure and ERG conditions have been reported previously [33,34,35,36]. Macular function was evaluated by multifocal ERG (LE-4100, Tomey, Nagoya, Japan), as previously described [37,38]. In the multifocal ERG system, the visual stimuli consist of 61 hexagonal elements with an overall subtense of approximately 50°.

### 2.4. Statistical Analysis

Statistical analyses were performed using IBM SPSS Statistics version 27.0 (IBM Corp, Armonk, NY, USA). The Bonferroni test was used to determine the significance of differences in the age at onset between phenotypes. Decimal BCVA was converted to logarithm of the minimum angle of resolution (logMAR) units for statistical analysis. The BCVA of counting fingers, hand motions, and light perception were converted to 2.0, 2.4, and 2.7 logMAR units, respectively [39]. Spearman’s rank correlation coefficient was used to evaluate the relationships between logMAR BCVA and age, and between the visual field areas of I-4e and V-4e isopters and age in patients with AR-RP. Kaplan–Meier survival curves with the log-rank test were used to compare survival experiences (in terms of logMAR BCVA and the visual field areas of I-4e and V-4e isopters) between patients with AR-RP and AD-RP. *p*-values <0.05 were considered statistically significant.

## 3. Results

### 3.1. Molecular Genetic Findings

**Figure 1:**
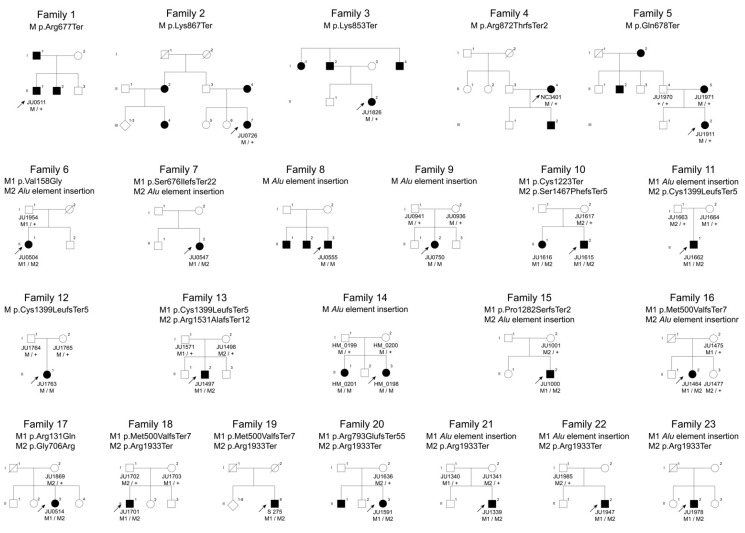
Pedigrees of the 23 Japanese families with *RP1*-associated retinal dystrophies. Five families (Families 1 to 5) with autosomal dominant retinitis pigmentosa (RP), 11 families (Families 6 to 16) with autosomal recessive RP and 7 families (Families 17 to 23) with autosomal recessive cone dystrophy/cone-rod dystrophy. Square boxes and circles indicate males and females, respectively. Filled symbols represent affected members, whereas unfilled symbols represent unaffected members. The plus sign denotes the wild-type allele, and the arrow indicates the proband of the family.

We identified 24 rare *RP1* variants in 38 patients from 36 families, including 18 pathogenic variants, with 11 known variants [c.1498_1499delAT, (p.Met500ValfsTer7), c.2029C > T, (p.Arg677Ter), c.2032C > T, (p.Gln678Ter), c.2116G > C, (p.Gly706Arg), c.2377delA, (p.Arg793GlufsTer55), c.2599A > T, (p.Lys867Ter), c.2613dupA, (p.Arg872ThrfsTer2), c.3669C > A, (p.Cys1223Ter), *Alu* element insertion, c.4196delG, (p.Cys1399LeufsTer5), and c.5797C > T, (p.Arg1933Ter)] and seven novel variants [c.392G > A, (p.Arg131Gln), c.473T > G, (p.Val158Gly), c.2020dupA, (p.Ser676IlefsTer22), c.2557A > T (p.Lys853Ter), c.3843dupT, (p.Pro1282SerfsTer2), c.4400delC, (p.Ser1467PhefsTer5), and c.4591_4592delAG, (p.Arg1531AlafsTer12)] from 23 families and six non-pathogenic variants [c.2400A > T, (p.Lys800Asn), c.2894G > T, (p.Ser965Ile), c.2951A > G, (p.Asp984Gly), c.2960G > A, (p.Cys987Tyr), c.3188A > G, (p.Gln1063Arg), c.5913C > A, (p.Asn1971Lys)] from 13 families (Figure 1 and Table 1). The six variants were “likely benign” or “uncertain significance” according to the ACMG and had inheritance inconsistencies.

#### 3.1.1. Autosomal-Dominant Retinitis Pigmentosa

In the five families with AD-RP (Families 1 to 5), five different heterozygous variants were identified, including four known *RP1* variants (p.Arg677Ter, p.Gln678Ter, p.Lys867Ter, and p.Arg872ThrfsTer2) and one novel variant (p.Lys853Ter). The novel variant (p.Lys853Ter) was determined as “likely pathogenic” according to the ACMG criteria. These five pathogenic variants were located within the hotspot region for AD-RP.

#### 3.1.2. Autosomal-Recessive Retinitis Pigmentosa

In the 11 families with AR-RP (Families 6 to 16), nine different variants were identified, including four known *RP1* variants (p.Met500ValfsTer7, p.Cys1223Ter, p.Cys1399LeufsTer5, and *Alu* element insertion) and five novel variants (p.Val158Gly, p.Ser676IlefsTer22, p.Pro1282SerfsTer2, p.Ser1467PhefsTer5, and p.Arg1531AlafsTer12). Of the five novel variants, the missense variant (p.Val158Gly) was considered as “likely pathogenic” according to the ACMG. Most pathogenic missense variants were located within the DCX domain or BIF region. The p.Val158Gly variant, which was identified in combination with *Alu* element insertion, was also located within the DCX domain. The remaining four novel variants, which were also identified in combination with reportedly pathogenic variants, were determined as “pathogenic” according to the ACMG criteria.

#### 3.1.3. Autosomal-Recessive Cone Dystrophy/Cone-Rod Dystrophy

In the seven families with AR-COD/CORD (Families 17 to 23), four kinds of compound heterozygous variants [(p.Arg131Gln/p.Gly706Arg), (p.Met500ValfsTer7/p.Arg1933Ter), (p.Arg793GlufsTer55/p.Arg1933Ter), and (*Alu* element insertion/p.Arg1933Ter)] were identified. Of these, the variant (p.Arg131Gln) was a novel missense variant; the other five variants (p.Met500ValfsTer7, p.Gly706Arg, p.Arg793GlufsTer55, *Alu* element insertion, and p.Arg1933Ter) have been reported as pathogenic variants [40,41,42,43]. The variant (p.Arg131Gln) was considered as “uncertain significance” according to the ACMG; however, we concluded that the variant was pathogenic because of its identification in combination with reportedly pathogenic variants, co-segregation analysis confirmation, phenotype consistency, and the possibility of a hypomorphic variant due to its location outside the DCX domain.

#### 3.1.4. Alu Element Insertion Analysis

**Figure 2:**
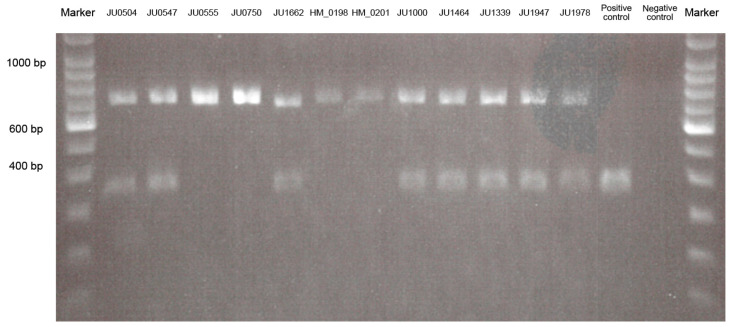
Results of the polymerase chain reaction-length polymorphism analysis for *Alu* element insertion.

The *Alu* element insertion was not always found by WES analysis. Therefore, we performed PCR-based screening for the *Alu* element insertion (Figure 2). The *Alu* element insertion was detected heterozygously in eight patients from eight families, including five families with AR-RP and three families with AR-COD/CORD, and homozygously in four patients from three families with AR-RP (Table 1 and Figure 2). The results revealed that the *Alu* element insertion was the most frequently observed pathogenic variant (32.0%, 16/50 alleles) in this study.

The 2% gel photograph shows the results for wild-type, heterozygous, and homozygous states in patients with autosomal recessive (AR)-retinitis pigmentosa (RP), patients with AR-cone dystrophy (COD)/cone-rod dystrophy (CORD), and controls. *Alu* element insertion is detected heterozygously in eight patients, including five patients (JU0504, JU0547, JU1662, JU1000, and JU1464) in AR-RP and three patients (JU1339, JU1947, and JU1978) in AR-COD/CORD, and homozygously in four patients (JU0555, JU0750, HM_0198, and HM_0201) with AR-RP. Polymerase chain reaction products indicate lengths of 347 bp in the wild-type allele and 675 bp in the *Alu* insertion allele.

### 3.2. Clinical Findings

We investigated 25 patients from 23 families with *RP1*-associated retinal dystrophies, including five patients with AD-RP from five families, 13 patients with AR-RP from 11 families, and seven patients with AR-COD/CORD from seven families (Figure 1). Clinical characteristics of the 25 patients are summarized in Supplemental Table S1. All 25 patients did not exhibit other systemic findings such as, hearing loss, psychomotor developmental delay, and polydactyly.

#### 3.2.1. Visual Acuity Assessment

**Figure 3:**
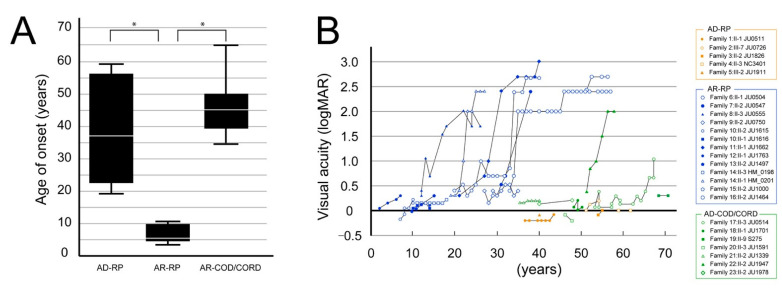
The age at onset and course of visual acuity findings are shown for the 3 phenotypes. (**A**) The graph shows the age at onset in patients with autosomal dominant (AD)-retinitis pigmentosa (RP), autosomal recessive (AR)-RP, and AR-cone dystrophy (COD)/cone-rod dystrophy (CORD). The age at onset significantly differed between patients with AR-RP and AD-RP (*p* < 0.001, Bonferroni test) and between patients with AR-RP and AR-COD/CORD (*p* < 0.001), but not between patients with AD-RP and AR-COD/CORD (*p* = 0.508). The asterisks indicate statistical significance (*p*-values < 0.05). (**B**) The graph shows the course of visual acuity in patients with AR-RP (in blue), AD-RP (in orange), and AR-COD/CORD (in green). Visual acuity starts to worsen in patients with AR-RP around their 20s and reaches severe visual dysfunction by their 40s; in contrast, good visual acuity is preserved in patients with AD-RP until their 50–60s. Furthermore, visual acuity shows a tendency toward relative preservation in patients with AR-COD/CORD until their 50s, with subsequent deterioration and progressive macular atrophy.

The age at onset and clinical course of visual acuity in the three phenotypes (AD-RP, AR-RP, and AR-COD/CORD) are shown in Figure 3. The age at onset was significantly earlier in patients with AR-RP (6.92 ± 0.82 years; range, 3–12 years) than in patients with AD-RP (39.8 ± 7.37 years; range, 18–59 years) and AR-COD/CORD (45.7 ± 4.76 years; range, 34–65 years) (*p* < 0.001); however, there was no difference in the age at onset between patients with AD-RP and AR-COD/CORD (*p* = 0.508) (Figure 3A). Visual acuity started to worsen in patients with AR-RP around their 20s and reached severe visual dysfunction by their 40s; in contrast, good visual acuity was preserved in patients with AD-RP until their 50–60s (Figure 3B). Furthermore, visual acuity showed a tendency toward relative preservation in patients with AR-COD/CORD until their 50s, with subsequent deterioration and progressive macular atrophy (Figure 3B).

#### 3.2.2. Visual Acuity and Visual Fields in Patients with Autosomal Recessive Retinitis Pigmentosa

**Figure 4:**
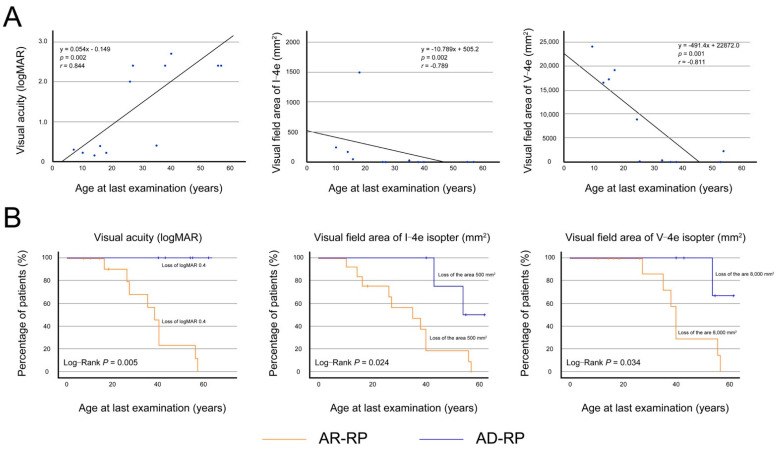
Visual acuity and visual field areas in patients with autosomal recessive retinitis pigmentosa. (**A**) In the left eyes (*n* = 13) of the 13 patients with autosomal recessive retinitis pigmentosa, the graphs show scatter plots of the logarithm of the minimum angle of resolution (logMAR) best-corrected visual acuity (BCVA) and visual field areas of I-4e and V-4e isopters as a function of the age at last examination. There was significant correlation between the BCVA and age (r = 0.844, *p* = 0.002, Spearman’s rank-order correlation), and between the visual field areas of I-4e and V-4e and age (r = −0.789, *p* = 0.002; r = −0.811, *p* = 0.001, respectively). Each graph indicates age-dependent deterioration, reaching to severe impairment around the 50s. (**B**) The graph shows the Kaplan–Meier survival curves, with log-rank tests, for visual acuity and visual field areas of I-4e and V-4e isopters in patients with autosomal recessive (AR)-retinitis pigmentosa (RP) and autosomal dominant (AD)-RP. The following cutoff points were used: BCVA ≤ 0.4 logMAR units (0.4 decimal units), I-4e isopter area ≤ 500 mm^2^ (10°), and V-4e isopter area ≤ 8000 mm^2^ (40°). Patients with AR-RP show significantly faster progression in the loss of visual acuity (*p* = 0.020) and visual field areas of I-4e (*p* = 0.011) and V-4e isopters (*p* = 0.024) in comparison to patients with AD-RP. The survival curves indicate that visual acuity and visual field areas are relatively preserved in most patients with AD-RP until their 40s but are severely impaired in most patients with AR-RP.

Data from the left eyes (*n* = 13) of the 13 AR-RP patients were used for statistical analysis because of the significant correlations between visual acuity of the right and left eyes (r = 0.943, *p* < 0.001) and the visual-field areas of I-4e (r = 1.000, *p* < 0.001) and V-4e (r = 0.952, *p* < 0.001). Figure 4A showed the relationships between logMAR BCVA and the visual field areas of I-4e and V-4e isopters and age at the last examination. The logMAR BCVA significantly deteriorated with age (r = 0.844, *p* = 0.002), and started to worsen in the 20s, reaching to light perception around the 50s. The visual field areas of I-4e and V-4e isopters also significantly deteriorated with age (r = −0.789, *p* = 0.002 and r = −0.811, *p* = 0.001), disappearing around the 50s.

Next, to compare the disease course of logMAR BCVA and visual field areas in patients with AR-RP and AD-RP, Kaplan–Meier survival curves were plotted with the following cut-offs: BCVA ≤ 0.4 logMAR (0.4 decimal units), I-4e isopter area ≤ 500 mm^2^ (10°), and V-4e isopter area ≤ 8000 mm^2^ (40°) (Figure 4B). Kaplan–Meier survival curve analysis with the log-rank test revealed that the progression in decreased visual acuity (*p* = 0.020) and loss of visual field areas of I-4e and V-4e isopters (*p* = 0.011; *p* = 0.024, respectively) were significantly faster in patients with AR-RP than in patients with AD-RP. The survival curves indicated that visual acuity and visual field areas were relatively preserved in most patients with AD-RP until their 40s but were severely impaired in most patients with AR-RP.

#### 3.2.3. Multimodal Retinal Imaging in Each Phenotype

**Figure 5:**
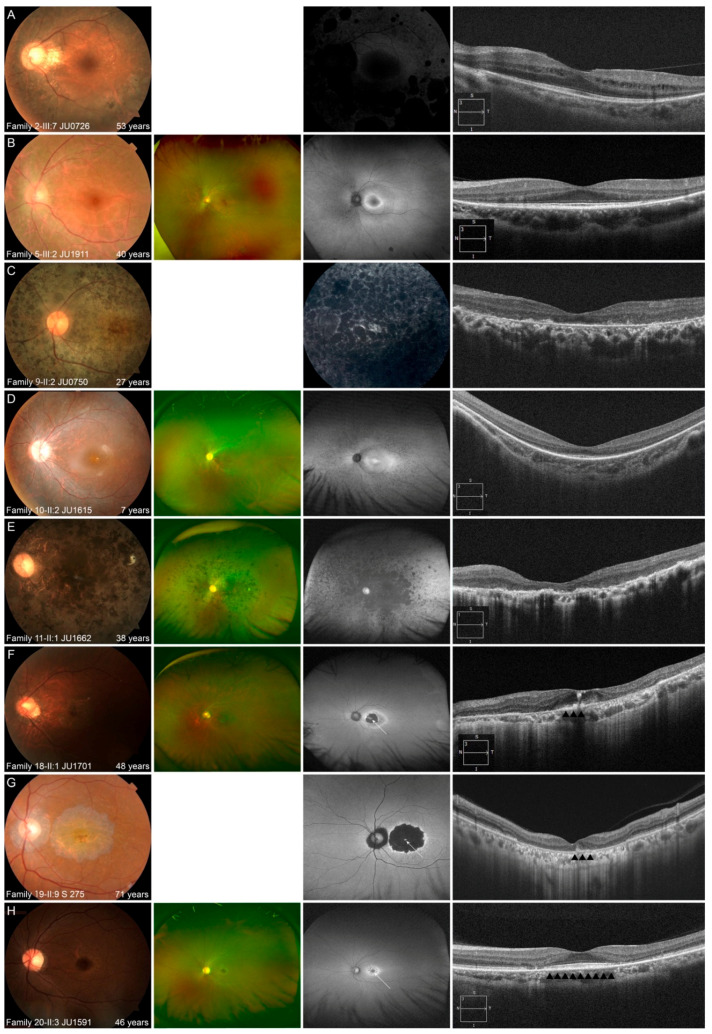
Representative multimodal retinal images are shown for the 3 phenotypes. (**A**,**B**) Multimodal retinal imaging of the left eye in 2 patients with autosomal dominant retinitis pigmentosa (RP) is shown. Fundus photography reveals retinal degeneration with/without pigmentation around arcade vessels (**A**,**B**). Fundus autofluorescence (FAF) reveals a normal appearance at the macular and hypo-autofluorescence (AF) corresponding to retinal degeneration and pigmentation (**A**), and ring-shaped hyper-AF at the macular and hypo-AF at the nasal retina (**B**). Optical coherence tomography (OCT) reveals no abnormalities of the outer retinal layers, including the fovea, with the exception of the nasal and temporal retina (**A**,**B**). (**C**–**E**) Multimodal retinal imaging of the left eye of 3 patients with autosomal recessive (AR)-RP is shown. Fundus photography reveals retinal degeneration not only at peripheral retina, but also at the macular, even in a younger patient (**C**–**E**). FAF reveals diffuse hypo-AF, including the fovea, in an older patient (**C**,**E**) and hyper-AF at the macular with hypo-AF at the peripheral retina in a younger patient (**D**). OCT reveals a blurred ellipsoid zone (EZ) at the fovea and disruption of almost all outer retinal layers, including the EZ, at other areas in a younger patient (**D**) and disruption of almost all outer retinal layers, including the EZ at the macular in older patients (**C**,**E**). (**F**–**H**) Multimodal retinal imaging of the left eye of 3 patients with AR cone dystrophy/cone-rod dystrophy is shown. Fundus photography reveals macular atrophy with foveal sparing (**F**–**H**). Ultra-widefield FAF reveals normal-AF at the preserved area (white arrow), with hypo-AF in the surrounding area (**F**); hyper-AF at the preserved area (white arrow) with hypo-AF in the surrounding area (**G**); and normal-AF at the preserved area (white arrow) with hyper-AF in the surrounding area (**H**). OCT reveals relatively preserved outer retinal layers including EZ at preserved area (black arrowheads) and disruption of almost all outer retinal layers, corresponding with macular atrophy (**F**,**G**) and no abnormalities of the outer retinal layers (black arrowheads), including the fovea, and disruption of almost all outer retinal layers, corresponding with macular atrophy (**H**).

Multimodal retinal imaging and ERG findings of representative cases are shown in Figure 5 and Supplemental Figures S1 and S2.

In the five patients with AD-RP, fundus photography revealed milder retinal degeneration, represented by slight retinal degeneration around arcade vessels with/without pigmentation and normal appearance, than that in patients with AR-RP. FAF/FA also revealed that retinal degeneration, especially macular, was milder than that in patients with AR-RP. OCT also revealed that the outer retinal layers, including the ellipsoid zone (EZ), was preserved at fovea, and the length of the preserved EZ tended to be longer than that in patients with AR-RP.

In the 13 patients with AR-RP, fundus photography revealed retinal degeneration not only at the (mid) peripheral retina, but also at the macular, even in younger patients. These characteristics were also observed on FAF, which showed diffusely hypo-AF or hyper-fluorescence at the posterior pole, including the fovea, in older patients, and hyper-AF at the macular, with/without hypo-AF at the peripheral retina, in younger patients. OCT revealed disruptive/blurred outer retinal layers, including the EZ at the fovea, even in younger patients. Patients with AR-RP were characterized by retinal degeneration in the peripheral retina, as well as in the macular, even in young patients, leading to severe retinal degeneration, including macular, in their 20′s.

In the seven patients with AR-COD/CORD, the common characteristic findings were macular atrophy with foveal sparing, normal/hyper-AF at the fovea with hyper/hypo-AF in the surrounding area, relatively preserved outer retinal layers, including the EZ and retinal pigment epithelium (RPE), corresponding to the area of foveal sparing, and disruption of almost all outer retinal layers with RPE thinning, corresponding to macular atrophy on OCT. Multifocal ERG findings in one patient (Family 20-II:3 JU1591) showed preserved responses in the central areas of both eyes (Supplemental Figure S2), and indicated findings of macular atrophy with foveal sparing. In older patients, macular atrophy was enlarged, and the area of foveal sparing became smaller on multimodal retinal imaging. ERG was performed in six patients and revealed that cone and rod functions tended to deteriorate with age (Supplemental Figure S1).

**Figure 6:**
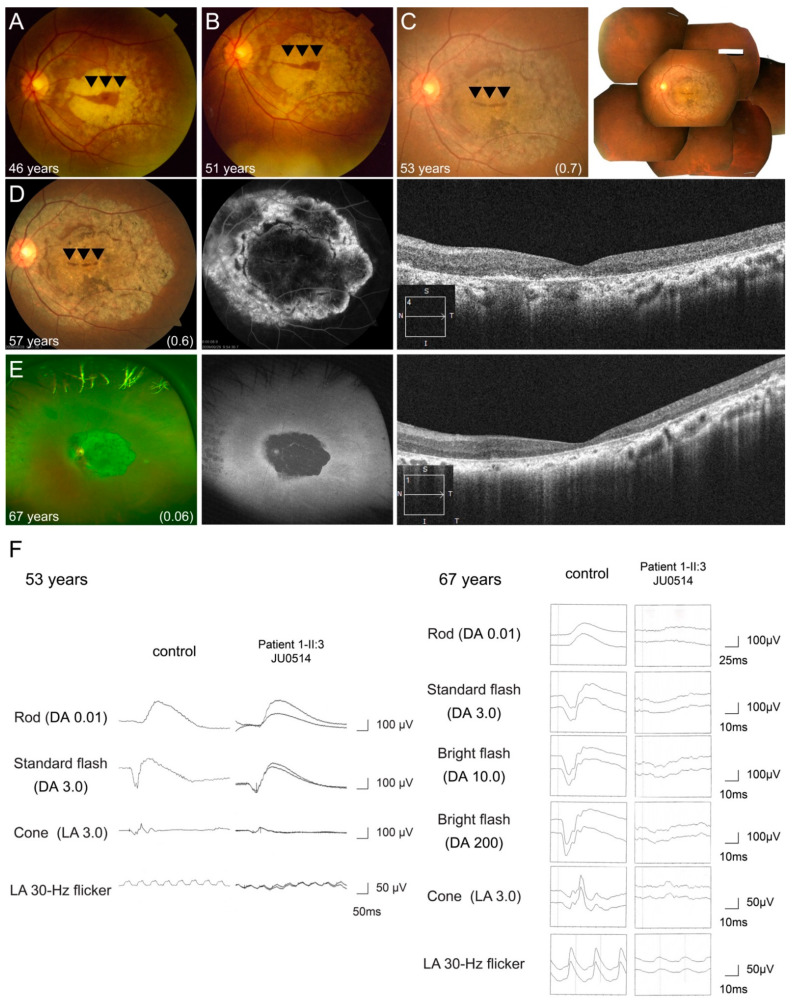
Longitudinal findings of a patient (Family 17-II:3 JU0514) with autosomal recessive cone-rod dystrophy. Multimodal retinal imaging findings of the left eye are shown at ages of 46, 51, 53, 57, and 67 years. Fundus photography shows macular atrophy with foveal sparing (arrowheads) at 46 years of age (**A**), and gradual enlargement toward not only the mid-peripheral retina, but also the fovea, resulting in an increasingly smaller area of foveal sparing (arrowheads) (**B**–**D**). At 67 years of age, retinal atrophy presented at the posterior pole and peripheral retina and the area of foveal sparing disappeared (**E**). Fundus autofluorescence and hypo-autofluorescence (AF) reveal hypo-AF within arcade vessels and hypo-fluorescence at the macular, with hyper-fluorescence surrounding the area, indicating severe retinal atrophy (**D**,**E**). OCT reveals progressive thinning of the outer retinal layers and retinal pigment epithelium, including the fovea (**D**,**E**). Full-field electroretinography (ERG) was performed at the ages of 53 and 67 years. ERG initially shows preserved rod responses and severely decreased cone responses (**F**). Fourteen years later, ERG reveals more progressive deterioration of rod responses than cone responses (**F**).

One patient (Family 17-II:3 JU0514) showed progressive cone and rod dysfunction during the 22-year follow-up period. Fundus photography showed macular atrophy with foveal sparing at 46 years of age (Figure 6A), and gradual enlargement toward not only the mid-peripheral retina, but also the fovea, resulting in an increasingly smaller area of foveal sparing (Figure 6B–D). At 67 years of age, retinal atrophy presented at the posterior pole and peripheral retina, and the area of foveal sparing disappeared (Figure 6E). Ultra-widefield FAF and FA revealed hypo-AF within arcade vessels and hypo-fluorescence at the macular, with hyper-fluorescence in the surrounding area, indicating severe retinal atrophy (Figure 6D,E). OCT also revealed progressive thinning of the outer retinal layers and RPE, including the fovea (Figure 6D,E). Furthermore, ERG at 53 years of age showed within normal range in the b-waves of DA 0.01, approximately 40% and 70% of our controls [35] in the a- and b-waves of DA 3.0, approximately 50% of the controls in the a- and b- waves of LA 3.0, and approximately 70% of the controls in the b-waves of LA 3.0 flicker. 14 years later, ERG revealed reduced to 10–15% of our controls [36] in the b-waves of DA 0.01 and the a- and b-waves of DA 3.0, 20% of the controls in the a- and b-waves of LA 3.0 and the b-waves of LA3.0 flicker (Figure 6F).

## 4. Discussion

Detailed genotype-phenotype correlations in *RP1*-associated retinal dystrophies had not been investigated in the Japanese population. In the present study, we described the clinical and genetic characteristics of 25 patients from 23 Japanese families with three different phenotypes (AD-RP, AR-RP, AR-COD/CORD), and 11 known and 7 novel variants were identified.

Genotype–phenotype correlations have been investigated in patients with *RP1* variants [14,18,44,45], and over 170 RP1 variants, including our 18 pathogenic variants, have been reported as causes of AR-RP, AD-RP, and AR-COD/CORD. In the AD-RP phenotype, all *RP1* truncated variants located within the hotspot region express truncated proteins, suggesting a dominant-negative effect mechanism [16,17]. Five variants identified in our study were also located within the hotspot region (Table 1). Unlike AD-RP, the genetic characteristics of AR-RP are not simple. Generally, AR-RP is mostly caused by biallelic truncated variants outside the hotspot region, leading to a loss of RP1 function [18]. A recent study on *Alu* element insertion reported that 5 of 26 (19%) patients with AR-RP with heterozygous *RP1* variants located outside the hotspot region had *Alu* element insertion in the other alleles, suggesting that *Alu* element insertion might be found in the other alleles in patients with AR-RP showing only heterozygous *RP1* variants [21]. However, 17 truncated variants have also been located within the hotspot region in patients with AR-RP [14,15,25,41,43,46,47,48]. These truncated variants might underlie the loss of function responsible for AR-RP [44,49]. In the current study, one of five patients with AR-RP carried the heterozygous *Alu* element insertion, with the p.Met500ValfsTer7 variant, located within the hotspot region, in the other allele (Family 16-II:2 JU1464) (Figure 1). However, the patient’s mother (Family 16-I:2 JU1475) was unaffected, consistent with AR inheritance. Furthermore, 10 missense variants, with 7 located within the DCX domain or BIF region, have also been associated with AR-RP [24,41,43,50,51,52,53,54,55,56,57]. Surprisingly, even some missense variants (p.Leu172Arg, p.Asp202Glu, p.Gly203Arg, and p.Phe227Val) located within the DCX domain in homozygous states have been reported as causes of AR-RP [50,52,54,58]. In the current study, we also identified compound heterozygous variants, consisting of *Alu* element insertion and the p.Val158Gly variant, located within DCX domain, in a patient with AR-RP (Family 6-II:1 JU0504). Collectively, these findings suggest that any type of rare variant in the *RP1* gene could have pathogenicity in patients with AR-RP.

The genetic characteristics of AR-COD/CORD are more complex than those of AD-RP and AR-RP. Previous studies have reported that functional hypomorphic variants (p.Phe180Cys, p.Val190Gly, and p.Arg1933Ter) in combination with pathogenic missense or truncated variants are associated with AR-COD/CORD [20,25]. Based on the current and previous studies, two genetic characteristics of the AR-COD/CORD phenotype are as follows: (1) homozygotes with the hypomorphic variant (p.Arg1933Ter) exhibit a normal phenotype, even at 80 years of age; [20] and (2) homozygous missense or truncated variants have never been identified in patients with AR-COD/CORD. To date, nine types of compound heterozygous variants, including our compound heterozygous variants (p.Arg131Gln/ p.Gly706Arg, p.Met500ValfsTer7/p.Arg1933Ter, Alu element insertion/p.Arg1933Ter, and p.Arg793GlufsTer55/p.Arg1933Ter), have been identified in 18 patients with AR-COD/CORD, including a hypomorphic variant combined with a reportedly pathogenic variant found in patients with AR-RP [14,25,43,58]. However, a few cases contradict these genetic characteristics of the AR-COD/CORD phenotype [20,48]. For example, AR-RP phenotypes have been reported even in the presence of a hypomorphic variant in one allele [48,50,59]. This phenotypic variability could be explained by the presence of additional genetic modifier variants other than the *RP1* gene, or the presence of a variant within the non-coding regions in *RP1*. Furthermore, as in Bardet–Biedl syndrome [60], the pathomechanism could show oligogenicity, in which two or more recessive genes variants may lead to IRD phenotypes when the variants act together [61]. Consistent with this, a previous study on *RP1*-associated retinal dystrophies indicated oligogenicity in 28 patients with the RP1 variant (p.Arg1933Ter) in one allele, in whom two identified *EYS* variants were significantly enriched [20]. This result suggests that the co-localization of EYS and RP1 proteins plays a role in cilial structure maintenance and the stabilization of disc membranes in the outer segment [62].

AD-RP has been reported to show a milder phenotype than AR-RP [19]. Consistent with this, we found a younger age at onset and faster disease progression in patients with AR-RP than in patients with AD-RP (Figure 3 and Figure 4). Furthermore, we focused on the time-to-central visual function loss in patients with AR-RP, revealing relatively preserved visual acuity and visual field areas in their 10s that started to worsen in their 20s, with a complete loss of visual function around their 50s (Figure 4). These results are supported by a recent study using adaptive optics that showed progressive macular involvement in patients with AR-RP, as well as age-dependent deterioration in preserved photoreceptor areas [22]. Thus, our study demonstrated that *RP1*-associated AR-RP exhibited early onset, severe and progressive visual impairment and seems to be one of the most severe forms of RP, indicating that promising early intervention will be needed to prevent the progression of vision loss in future. Regarding the AR-COD/CORD phenotype, it can be characterized as follows: late onset, initial bull’s eye maculopathy with foveal sparing, and progression to macular atrophy with foveal involvement [25]. In the current study, the seven patients with AR-COD/CORD had clinical findings similar to these reported characteristics. Furthermore, these characteristics were observed in a patient who underwent longitudinal observation (Family 17-III:3 JU0514), which revealed an initial presence of macular atrophy with foveal sparing, with over 20 years required for the disappearance of the preserved area, and progressive degeneration not only at retina, but also at the RPE (Figure 5). Considering the results of previous studies and our study, changes in the retinal structure in patients with AR-COD/CORD are likely to be similar to those in central areolar choroidal dystrophy, in terms of macular atrophy with foveal sparing, even partially in older patients, and the presence of degeneration in the RPE and outer retinal layers. Regarding retinal function, we found varying degrees of cone and rod dysfunction, represented by macular dystrophy, COD, and CORD, and a tendency toward age-dependent deterioration in cone and rod function (Supplemental Figure S1). Interestingly, longitudinal observations in one patient (Family 17-III:3 JU0514) reveled a COD phenotype at 53 years of age, followed by a predominant progression in rod function, resulting in CORD (Figure 6). The ERG finding of little progression in cone function over a 20-year period might be consistent with the genetic characteristics of AR-COD/CORD with a hypomorphic variant in one allele.

The present study has a few limitations; namely, the small number of patients and the fact that they were recruited from only four institutions. Further studies with a large number of patients would provide more strength for the evidence obtained in our clinical and genetic findings.

## 5. Conclusions

We described the clinical and genetic characteristics of 25 Japanese patients with *RP1*-associated retinal dystrophies, in whom seven novel variants were identified. Our results suggest a genotype–phenotype correlation between variant types/locations and phenotypes (AD-RP, AR-RP, and AR-COD/CORD). The *Alu* element insertion was the most frequently observed pathogenic variant (32.0%, 16/50 alleles) in our Japanese patients with *RP1*-associated retinal dystrophies. Our results have expanded the genetic spectrum and clinical profiles of *RP1*-associated retinal dystrophies.

## Figures and Tables

**Table 1:** Genetic findings of 23 Japanese families with pathogenic *RP1* variants and 13 families with non-pathogenic *RP1* variants.

Family ID Patient ID/Affeceted Number	Nucleotide Change Protein Change	Zygosity	Classification	Phenotype	dP SNP ID	Frequency in Database (%)			ACMG	Known Novel
						HGVD	ToMMo	gnomAD	Classification Criteria	
Family 1 JU0511/1	c.2029C > T (p.Arg677Ter)	Hetero	Exon 4 Class II	AD-RP	rs104894082	NR	NR	NR	Likely pathogenic (PM, PM2, PM4, PP3, PP4, PP5)	Known
Family 2 JU0726/1	c.2599A > T (p.Lys867Ter)	Hetero	Exon 4 Class II	AD-RP	NR	NR	NR	NR	Likely pathogenic (PM, PM2, PM4, PP3, PP4, PP5)	Novel
Family 3 JU1826/1	c.2557A > T (p.Lys853Ter)	Hetero	Exon 4 Class II	AD-RP	NR	NR	NR	NR	Likely pathogenic (PM1, PM2, PM4, PP4)	Known
Family 4 NC3401/1	c.2613dupA (p.Arg872ThrfsTer2)	Hetero	Exon 4 Class II	AD-RP	rs1449723475	NR	NR	NR	Likely pathogenic (PM1, PM2, PM4, PP3, PP4, PP5)	Known
Family 5 JU1911/1	c.2032C > T (p.Gln678Ter)	Hetero	Exon 4 Class II	AD-RP	rs878853328	NR	NR	0.000	Likely pathogenic (PM1,PM2, PM4, PP3, PP4, PP5)	Novel
Family 6 JU0504/1	c.473T > G (p.Val158Gly)	Compound hetero	Exon 2 Class I	AR-RP	NR	NR	NR	NR	Likely pathogenic (PM1, PM2, PM3, PP3)	Known
	c.4052_4053ins328 (p.Tyr1352AlafsTer9)		Exon 4 Class III		rs775253277	NR	NR	NR	Pathogenic (PVS1, PM2, PM4, PP3)	Novel
Family 7-JU0547/1	c.2020dupA (p.Ser676IlefsTer22)	Compound hetero	Exon 4 Class II	AR-RP	NR	NR	NR	NR	Pathogenic (PVS1, PM2, PM3, PM4)	Known
	c.4052_4053ins328 (p.Tyr1352AlafsTer9)		Exon 4 Class III		rs775253277	NR	NR	NR	Pathogenic (PVS1, PM2, PM4, PP3)	Known
Family 8 JU0555/1	c.4052_4053ins328 (p.Tyr1352AlafsTer9)	Homo	Exon 4 Class III	AR-RP	rs775253277	NR	NR	NR	Pathogenic (PVS1, PM2, PM4, PP3)	Known
Family 9 JU0750/1	c.4052_4053ins328 (p.Tyr1352AlafsTer9)	Homo	Exon 4 Class III	AR-RP	rs775253277	NR	NR	NR	Pathogenic (PVS1, PM2, PM4, PP3)	Known
Family 10 JU1615, JU1616/2	c.3669C > A (p.Cys1223Ter)	Compound hetero	Exon 4 Class II	AR-RP	rs765129639	NR	0.000	0.000	Pathogenic (PVS1, PM2, PM4, PP1, PP3, PP5)	Novel
	c.4400delC (p.Ser1467PhefsTer5)		Exon 4 Class III		NR	NR	NR	NR	Pathogenic (PVS1, PM2, PM3, PM4, PP1)	Known
Family 11 JU1662/1	c.4052_4053ins328 (p.Tyr1352AlafsTer9)	Compound hetero	Exon 4 Class III	AR-RP	rs775253277	NR	NR	NR	Pathogenic (PVS1, PM2, PM4, PP3)	Known
	c.4196delG (p.Cys1399LeufsTer5)		Exon 4 Class III		rs762951570	NR	0.000	0.000	Pathogenic (PVS1, PM2, PM3, PM4, PP5)	Known
Family 12-JU1763/1	c.4196delG (p.Cys1399LeufsTer5)	Homo	Exon 4 Class III	AR-RP	rs762951570	NR	0.000	0.000	Pathogenic (PVS1, PM2, PM4, PP5)	Known
Family 13- JU1497/1	c.4196delG (p.Cys1399LeufsTer5)	Compound hetero	Exon 4 Class III	AR-RP	rs762951570	NR	0.000	0.000	Pathogenic (PVS1, PM2, PM4, PP5)	Novel
	c.4591_4592delAG (p.Arg1531AlafsTer12)		Exon 4 Class III		NR	NR	NR	NR	Pathogenic (PVS1, PM2, PM3, PM4)	Known
Family 14 HM_0198, HM_0201/2	c.4052_4053ins328 (p.Tyr1352AlafsTer9)	Homo	Exon 4 Class III	AR-RP	rs775253277	NR	NR	NR	Pathogenic (PVS1, PM2, PM4, PP3)	Novel
Family 15 JU1000/1	c.3843dupT (p.Pro1282SerfsTer2)	Compound hetero	Exon 4 Class III	AR-RP	NR	NR	NR	NR	Pathogenic (PVS1, PM2, PM3, PM4)	Known
	c.4052_4053ins328 (p.Tyr1352AlafsTer9)		Exon 4 Class III		rs775253277	NR	NR	NR	Pathogenic (PVS1, PM2, PM4, PP3)	Known
Family 16 JU1464/1	c.1498_1499delAT (p.Met500ValfsTer7)	Compound hetero	Exon 4 Class II	AR-RP	rs765129639	NR	0.000	0.000	Pathogenic (PVS1, PM2, PM3, PM4, PP3, PP5)	Known
	c.4052_4053ins328 (p.Tyr1352AlafsTer9)		Exon 4 Class III		rs775253277	NR	NR	NR	Pathogenic (PVS1, PM2, PM4, PP3)	Novel
Family 17 JU0514/1	c.392G>A (p.Arg131Gln)	Compound hetero	Exon 2 Class I	AR-COD/ CORD	rs752150870	0.002	0.003	0.000	Uncertain Significance (PM2, PM3, PP3)	Known
	c.2116G > C (p.Gly706Arg)		Exon 4 Class II		rs199879316	0.000	0.000	0.000	Uncertain Significance (PM2, PP5)	Known
Family 18 JU1701/1	c.1498_1499delAT (p.Met500ValfsTer7)	Compound hetero	Exon 4 Class II	AR-COD/ CORD	rs765129639	NR	0.000	0.000	Pathogenic (PVS1, PM2, PM3, PM4, PP3, PP5)	Known
	c.5797C > T (p.Arg1933Ter)		Exon 4 Class IV		rs118031911	0.003	0.003	0.000	Pathogenic (PVS1, PM2, PM3, PM4)	Known
Family 19 S275/1	c.1498_1499delAT (p.Met500ValfsTer7)	Compound hetero	Exon 4 Class II	AR-COD/ CORD	rs765129639	NR	0.000	0.000	Pathogenic (PVS1, PM2, PM3, PM4, PP3, PP5)	Known
	c.5797C > T (p.Arg1933Ter)		Exon 4 Class IV		rs118031911	0.003	0.003	0.000	Pathogenic (PVS1, PM2, PM3, PM4)	Known
Family 20 JU1591/1	c.2377delA (p.Arg793GlufsTer55)	Compound hetero	Exon 4 Class II	AR-COD/ CORD	NR	NR	NR	NR	Pathogenic (PVS1, PM2, PM3, PM4)	Known
	c.5797C > T (p.Arg1933Ter)		Exon 4 Class IV		rs118031911	0.003	0.003	0.000	Pathogenic (PVS1, PM2, PM3, PM4)	Known
Family 21 JU1339/1	c.4052_4053ins328 (p.Tyr1352AlafsTer9)	Compound hetero	Exon 4 Class III	AR-COD/ CORD	rs775253277	NR	NR	NR	Pathogenic (PVS1, PM2, PM3, PM4, PP3)	Known
	c.5797C > T (p.Arg1933Ter)		Exon 4 Class IV		rs118031911	0.003	0.003	0.000	Pathogenic (PVS1, PM2, PM3, PM4)	Known
Family 22 JU1947/1	c.4052_4053ins328 (p.Tyr1352AlafsTer9)	Compound hetero	Exon 4 Class III	AR-COD/ CORD	rs775253277	NR	NR	NR	Pathogenic (PVS1, PM2, PM3, PM4, PP3)	Known
	c.5797C>T (p.Arg1933Ter)		Exon 4 Class IV		rs118031911	0.003	0.003	0.000	Pathogenic (PVS1, PM2, PM3, PM4)	Known
Family 23 JU1978/1	c.4052_4053ins328 (p.Tyr1352AlafsTer9)	Compound hetero	Exon 4 Class III	AR-COD/ CORD	rs775253277	NR	NR	NR	Pathogenic (PVS1, PM2, PM3, PM4, PP3)	Known
	c.5797C > T (p.Arg1933Ter)		Exon 4 Class IV		rs118031911	0.003	0.003	0.000	Pathogenic (PVS1, PM2, PM3, PM4)	Known
Family 24 JU1672/1	c.5913C > A (p.Asn1971Lys)	Hetero	Exon 4 Class IV	AD-RP	rs754290174	0.005	0.005	0.000	Likely benign (PM2, BP1, BP4)	Novel
Family 25 JU0518/1	c.5913C > A (p.Asn1971Lys)	Hetero	Exon 4 Class IV	AD-RP	rs754290174	0.005	0.005	0.000	Likely benign (PM2, BP1, BP4)	Novel
Family 26 JU0616/1	c.3188A > G (p.Gln1063Arg)	Hetero	Exon 4 Class III	AD-RP	rs199550930	NR	NR	0.000	Likely benign (PM2, BP1, BP4)	Novel
Family 27 JU0523/1	c.5797C > T (p.Arg1933Ter)	Hetero	Exon 4 Class IV	AR-RP	rs118031911	0.003	0.003	0.000	Pathogenic (PVS1, PM2, PM3, PM4)	Known
Family 28 JU0525/1	c.2400A > T (p.Lys800Asn)	Hetero	Exon 4 Class II	AR-RP	NR	NR	NR	NR	Uncertain Significance (PM2, BP1)	Novel
Family 29 JU0553/1	c.2951A > G (p.Asp984Gly)	Hetero	Exon 4 Class II	AR-RP	rs200135800	0.003	0.005	0.000	Likely benign (PM2, PP5, BP1, BP4)	Known
Family 30 JU1791/1	c.2400A > T (p.Lys800Asn)	Hetero	Exon 4 Class II	AR-RP	NR	NR	NR	NR	Uncertain Significance (PM2, BP1)	Novel
Family 31 JU1942/1	c.5797C > T (p.Arg1933Ter)	Hetero	Exon 4 Class IV	AR-RP	rs118031911	0.003	0.003	0.000	Pathogenic (PVS1, PM2, PM3, PM4)	Known
Family 32 JU0565/1	c.5797C > T (p.Arg1933Ter)	Hetero	Exon 4 Class IV	AR-COD/ CORD	rs118031911	0.003	0.003	0.000	Pathogenic (PVS1, PM2, PM3, PM4)	Known
Family 33 JU0632/1	c.5797C > T (p.Arg1933Ter)	Hetero	Exon 4 Class IV	AR-COD/ CORD	rs118031911	0.003	0.003	0.000	Pathogenic (PVS1, PM2, PM3, PM4)	Known
Family 34 JU0830/1	c.2960G > A (p.Cys987Tyr)	Hetero	Exon 4 Class II	AR-COD/ CORD	rs747536867	0.000	0.001	0.000	Likely benign (PM2, BP1, BP4)	Novel
Family 35 JU1191/1	c.2951A > G (p.Asp984Gly)	Hetero	Exon 4 Class II	AR-COD/ CORD	rs200135800	0.003	0.005	0.000	Likely benign (PM2, PP5, BP1, BP4)	Known
Family 36 JU1955/1	c.2894G > T (p.Ser965Ile)	Hetero	Exon 4 Class II	AR-COD/ CORD	rs201110322	0.042	0.040	0.005	Likely benign (PM2, BP1, BP4, BP6)	Novel

ACMG = the American College of Medical Genetics and Genomics, AD = autosomal dominant, AR = autosomal recessive, COD = cone dystrophy, Compound hetero=Compound heterozygous, CORD = cone-rod dystrophy, Hetero = heterozygous, Homo = homozygous, NR = not reported, db SNP, Single Nucleotide Polymorphism Database (https://www.ncbi.nlm.nih.gov/snp/, accessed on 1 June 2020); Gnom AD, Genome Aggregation Database (http://gnomad.broadinstitute.org, accessed on 1 October 2020), HGVD, Human Genetic Variation Database (http://www.hgvd.genome.med.kyoto-u.ac.jp/index.html, accessed 1 January 2020), ToMMo, The Tohoku Medical Megabank Organization of Tohoku University (https://ijgvd.megabank.tohoku.ac.jp, accessed on 1 February 2021).

## Data Availability

The imaging data are not publicly available due to their information that could compromise the patients’ privacy. The data in graphs and tables that support the findings of this study are available from the corresponding author upon reasonable request.

## Supplementary Materials

Supplementary materials can be found at https://www.mdpi.com/article/10.3390/jcm10112265/s1, Supplemental Figure S1: Representative full-field electroretinography findings of patients with pathogenic *RP1* variants, Supplemental Figure S2: Multifocal electroretinographic findings in a patient (Family 20-II:3 JU1591) with autosomal recessive cone-rod dystrophy, and Supplemental Table S1: Clinical findings of patients with pathogenic *RP1*. Supplemental Table S1: Clinical findings of patients with pathogenic *RP1* variants.
