# Supplementary figures and images for: Genotype-Phenotype Correlations in RP1-Associated Retinal Dystrophies: A Multi-Center Cohort Study in JAPAN
Source: J Clin Med. 2021 May 24;10(11):2265. doi: 10.3390/jcm10112265 (PMC8197273; doi:10.3390/jcm10112265)

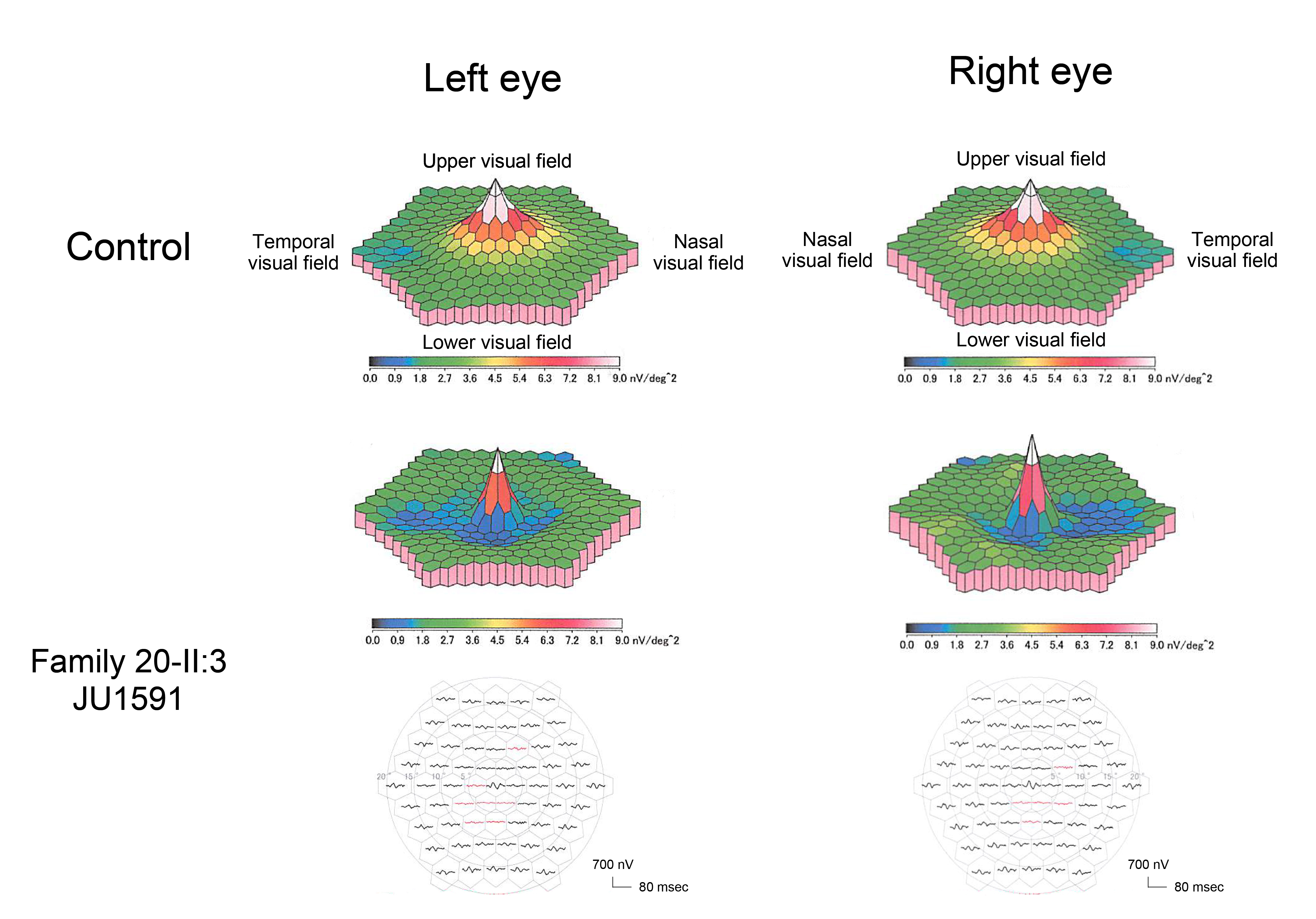

Supplement: Supplementary file 1 [file jcm-10-02265-s001.zip › jcm-1206736-supplementary 15.12.14/Supplemental Figure S2 Multifocal electroretinographic findings in a patient (Family 20-II3 JU1591) with autosomal re-cessive cone-rod dystrophy.jpg]
